# Improvement of *Mycoplasma pneumoniae*–Associated Acute Cerebellar Ataxia and Possible Encephalopathy After Intravenous Immunoglobulin

**DOI:** 10.1155/crdi/2971853

**Published:** 2026-03-09

**Authors:** Kathleen Ruff, Miltiadis Douvoyiannis

**Affiliations:** ^1^ University of North Dakota School of Medicine and Health Sciences, Grand Forks, North Dakota, USA, und.edu; ^2^ Pediatric Hospital Medicine and Pediatric Infectious Diseases Service, Altru Health System, Grand Forks, North Dakota, USA

**Keywords:** acute postinfectious cerebellar ataxia, encephalopathy, IVIG, *Mycoplasma pneumoniae*

## Abstract

*Mycoplasma pneumoniae* (*M. pneumoniae*) can cause acute postinfectious cerebellar ataxia and encephalitis/encephalopathy. Although ataxia can be self‐limited, prolonged duration of symptoms and long‐term neurological sequelae that persist for months or more are associated with both conditions. Multiple mechanisms have been suggested in the pathogenesis, but it seems that immune‐mediated damage plays a pivotal role. In this report, two children with community‐acquired pneumonia developed acute cerebellar ataxia (one with possible encephalopathy) associated with serologically confirmed *M. pneumoniae,* after having received antibiotics effective against *M. pneumoniae*. They improved rapidly after the administration of intravenous immunoglobulin (IVIG), without any sequelae. The exact role of immunomodulatory treatment such as IVIG in decreasing the severity and duration of *M. pneumoniae* acute cerebellar ataxia and encephalitis/encephalopathy and the criteria for administration need to be further studied.

## 1. Introduction

*Mycoplasma pneumoniae* (*M. pneumoniae*) is a common cause of community‐acquired pneumonia in children, accounting for 40%, and during epidemics, up to 70% of cases [1]. Additionally, it has been associated with extrapulmonary manifestations, the most often of which are central nervous system (CNS) conditions that may occur in 7%–11.5% of hospitalized children with *M. pneumoniae* infections [2–4]. These CNS manifestations include encephalitis/encephalopathy, aseptic meningitis, acute disseminated encephalomyelitis, transverse myelitis, Guillain–Barre syndrome, and acute cerebellar ataxia [5]. Suggested pathophysiologic mechanisms include direct CNS invasion, immune‐mediated damage by cross‐reacting autoantibodies, neurotoxin‐mediated injury, and immune‐complex vasculitis or vascular thrombi [5, 6]. The management of CNS manifestations associated with *M. pneumoniae* in children includes antibiotic therapy, immunosuppressant therapy such as steroids, and immunomodulatory agents such as intravenous immunoglobulin (IVIG), but there is no agreement on optimal management. This report describes the improvement of two children who had acute cerebellar ataxia (one with encephalopathy as well) attributed to *M. pneumoniae* after the administration of IVIG. Approval by the institutional review board was waived for this report.

## 2. Case Presentation

### 2.1. Case 1

A previously healthy, immunized 7‐year‐old girl was admitted because of a 2‐day loss of balance, difficulty walking, nausea, and vomiting of acute onset. Her mother described her as walking as though she was intoxicated. She had no fever, altered mental status, headaches, respiratory distress, diarrhea, chorea, or convulsions. She had been treated for streptococcal pharyngitis 3 weeks ago with amoxicillin for 10 days and for community‐acquired pneumonia by chest X‐ray 2 weeks ago with azithromycin for 5 days. She took no other medication and had no known allergies.

On physical examination, her temperature was 36.3°C, heart rate was 94 beats/min, respiratory rate was 16 breaths/min, oxygen saturation was 96%, and blood pressure was 109/60 mmHg. Her mental status was intact, and she exhibited no meningeal signs. Horizontal nystagmus was present. She had a positive heel‐to‐shin and finger‐to‐nose test. Her gait was severely wide‐based and ataxic that prevented her from independent ambulation. Her muscle strength and reflexes were normal. Her sensation was intact. Cardiorespiratory examination was normal. The rest of the physical examination was unremarkable.

A complete blood count (CBC) showed 9800 white blood cells/mcl with 55% neutrophils and 37% lymphocytes. Electrolytes and transaminases were within normal limits. C‐reactive protein was 1.1 mg/dL (normal< 0.5). She was treated with IVIG 500 mg/kg/day for 3 days and dexamethasone 2 mg IV prior to each infusion. After the first 2 doses, she had improved dysmetria and gait ataxia. On the third day, she was discharged home with a cane. Her symptoms resolved 3 weeks later.

### 2.2. Case 2

A previously healthy, immunized 10‐year‐old girl was admitted with a one‐day history of sudden onset wide‐based gait and difficulty with ambulation since she woke up. She had been having recurrent vomiting, lightheadedness, and a feeling of things spinning around her for 2 days prior to admission. She had been receiving doxycycline for 7 days for community‐acquired pneumonia. On physical examination, she was afebrile; her heart rate was 72 beats/min, respiratory rate was 18 breaths/min, oxygen saturation was 97%, and blood pressure was 98/57 mmHg. She was somnolent but arousable and oriented. She exhibited no meningeal signs. Horizontal nystagmus was noted. She had gait and truncal ataxia with dysmetria and dysdiadochokinesia. She was unable to stand. Muscle strength, sensation, and deep tendon reflexes were normal. Left lower lung rales were detected, but the remainder of the examination was normal.

A CBC showed 10,800 white blood cells/mcl with 84% neutrophils and 13% lymphocytes. Electrolytes, transaminases, C‐reactive protein, procalcitonin, serum ammonia, and urine drug screen testing were normal.

She was treated with IVIG 500 mg/kg/day for 3 days and methylprednisolone 0.25 mg/kg/dose IV prior to each infusion. After the first 2 doses of IVIG, she had improved mental status, dysmetria and gait ataxia. On the third day she was discharged home able to ambulate without any walking assistive device. Her symptoms had resolved 7 days later.

Both children had the following tests on admission: a negative respiratory pathogen polymerase‐chain reaction (PCR) panel testing on nasopharyngeal swab using the FilmArray Respiratory Panel 2.1 assay (BioFire Diagnostics). Agents detected by this panel include coronaviruses, influenza A and B, parainfluenza 1, 2, and 3, adenovirus, respiratory syncytial virus, metapneumovirus, rhinovirus and/or enterovirus, *M. pneumoniae, Chlamydia pneumoniae, Bordetella Pertussis*, and *Bordetella parapertussis*. Also, they had a negative serology for Epstein–Barr virus. *M. pneumoniae* was not detected by PCR on a specimen obtained by an oropharyngeal swab. Screening serology for *M. pneumoniae* was positive for IgM and IgG antibodies by enzyme immunoassay (EIA), and IgM positivity was verified by indirect immunofluorescence assay (IFA).

Both had a normal computed tomography (CT) scan and magnetic resonance imaging (MRI) of the brain, and a repeat chest X‐ray that showed near resolution of their pneumonia. Neither child had a lumbar puncture performed.

## 3. Discussion

In this report, two children developed acute cerebellar ataxia (Case 2 with possible encephalopathy) attributed to *M. pneumoniae*, although they had received antibiotics against *M. pneumoniae*. The administration of IVIG ameliorated their clinical symptoms and signs within 2 days suggesting an underlying immune‐mediated process. Although positive *M. pneumoniae* IgM may persist for months after an acute infection, the fact that the pneumonia in the two presented cases responded to azithromycin and doxycycline, and that during that period there was an outbreak of *M. pneumoniae* in children in our community, is supportive of the hypothesis that *M. pneumoniae* was associated with the development of CNS complications in this report. Additionally, the positive IgM was verified by indirect IFA. In both children, *M. pneumoniae* was not detected by PCR, which may be related to the prior administration of active antibiotics against *M. pneumoniae*.

Over a 24‐year period, 45 children were hospitalized with encephalitis associated with serologically confirmed *M. pneumoniae*. The mean hospital stay was 2–3 weeks [2]. Among 365 children hospitalized with *M. pneumoniae* infection detected by PCR, neurologic diseases developed in 42 (11.5%) [4]. Encephalitis/encephalopathy was detected in 52% of them and cerebellar ataxia in 10%. In cases of encephalitis, a mild lymphocytic pleocytosis and elevation of the protein may be detected in the cerebrospinal fluid (CSF) [2, 3, 7]. Electroencephalography is usually abnormal, but brain MRI may not detect any lesions [2, 3]. Long‐term sequelae, such as cognitive impairments, seizures, and movement disorders, have been reported in 23%–64% of patients with *M. pneumoniae* encephalitis [2, 3]. In a large series, half of the children with *M. pneumoniae* encephalitis suffered neurologic sequelae, although they received antibiotic therapy. Moreover, 71% of those who received steroids did not fully recover [4]. This data show the need for additional or alternative interventions in children with *M. pneumoniae* encephalitis/encephalopathy.

Acute postinfectious cerebellar ataxia is characterized by acute truncal and gait ataxia, with additional possible signs of dysmetria, nystagmus, and hypotonia [8]. Infectious agents associated with acute postinfectious cerebellar ataxia include varicella, measles, mumps, rubella, Epstein–Barr virus, enteroviruses, herpes simplex type 1, influenza, parvovirus B19, *M. pneumoniae*, and *Bordetella pertussis*, among others [8, 9]. Acute postinfectious cerebellar ataxia can be self‐resolving in 2–3 weeks [10]. Nevertheless, it may last on average 2 months and may persist even 6 months later [11, 12]. Additionally, severe gait ataxia—not being able to walk as in our patients—has been correlated with a more prolonged recovery (*p* < 0.0004) [11].

A more severe inflammatory presentation is cerebellitis, characterized additionally by acute intracranial pressure elevation, manifesting with headache, vomiting, and altered consciousness/somnolence, in which brain MRI may be abnormal and has a more severe clinical course [8, 9]. Neurologic sequelae developed in 4 of 15 children (27%) and in 3 of 9 children (33%) after acute cerebellitis [8, 9].

Early‐onset encephalitis that occurs within 7 days of the onset of a prodromal respiratory syndrome caused by *M. pneumoniae* is associated with a more frequent detection of *M. pneumoniae* DNA by PCR in the CSF and is considered a result of direct CNS invasion [4]. Conversely, when encephalitis occurs after 7 days from the onset of the prodrome, an immunologically based mechanism is implicated. The children in this report had development of CNS disease after the first 7 days of pneumonia, which justified the administration of IVIG. Steroids were administered as well but at much lower dosages as reported in the literature for similar conditions.

Others believe that there may be an overlap of the pathophysiologic mechanisms suggested to cause CNS diseases associated with *M. pneumoniae* [6]. One kind of treatment may not be enough, and a combination of medications may be necessary.

Nevertheless, it is reasonable to administer antibiotic therapy regardless of the type of CNS disease and the presumed pathogenesis [3, 7]. When choosing an antibiotic, the penetration of the blood–brain barrier should be considered. Tetracyclines such as doxycycline and fluoroquinolones such as levofloxacin have better penetration into the CNS than azithromycin [6]. Another factor to be considered is the incidence of macrolide‐resistant *M. pneumoniae* in the community.

The first patient in this report had community‐acquired pneumonia that had resolved after azithromycin administration. So, it was considered that CNS direct invasion should have been covered by that administration and the subsequent acute cerebellar ataxia was caused by immune dysregulation. The second patient was being treated for pneumonia with doxycycline, as an increased incidence of macrolide‐resistant *M. pneumoniae* was observed in our community [13]. The American Academy of Pediatrics has allowed the use of tetracyclines in children < 8 years old for a shorter period of < 21 days, considering that there is no risk of permanent dental staining according to accumulating data [14].

There is a limited number of reports regarding the efficacy of IVIG administration in ataxia and encephalitis/encephalopathy caused by *M. pneumoniae*. A 4‐year‐old boy who developed acute cerebellar ataxia with dysmetria for a month and who failed steroid treatment had resolution of his symptoms within a week after IVIG administration (400 mg/kg/day for 5 days) [15]. A 6‐year‐old girl with *M. pneumoniae* pneumonia who developed encephalitis, improved within 48 h of IVIG administration (500 mg/kg/daily for 3 days) [16]. Four of nine children with acute cerebellitis received IVIG (1–3 courses) in combination with steroids as part of their treatment, but half of them had long‐term complications [8]. Four children with *M. pneumoniae* encephalitis/encephalopathy improved after IVIG administration (1 gr/kg/day for 2 days or 500 mg/kg/day for 3 days) and 3 of them promptly [17]. A 12‐year‐old girl who developed acute cerebellar ataxia and encephalitis attributed to *M. pneumoniae*, exhibited improvement within 48 h of the administration of IVIG (1 gr/kg/day for 3 days) [18]. Conversely, in a series of 15 children with acute cerebellitis, 8 received IVIG, but the authors did not observe any significant improvement after that [9]. A multicenter study detected that among 87 children with *M. pneumoniae* encephalitis who received azithromycin, those who received additional IVIG (42%) (400 mg/kg/day for 5 days or 1 gr/kg/day for 2 days) had a significant better clinical course and shorter duration of hospitalization [7].

Acute postinfectious cerebellar ataxia may be self‐resolving. Nevertheless, we proceeded with the administration of IVIG for the following reasons: no improvement was noticed in the first child after 3 days; additional manifestations occurred such as dysmetria and nystagmus (both cases), somnolence with recurrent vomiting and vertigo (Case 2), and severe ataxia with nonambulation was exhibited (both cases), which has been associated with more prolonged recovery [11]. More than 7 days had passed since the preceding pneumonia that had responded to antibiotics, implicating an immunologic pathogenetic mechanism of the CNS manifestations. Additionally, there are no well‐defined and studied prognostic factors that can predict clinical deterioration among children with *M. pneumoniae* acute cerebellar ataxia or encephalitis/encephalopathy. Brain MRI may be normal in more than half of cases of *M. pneumoniae* encephalitis/encephalopathy [3] and is not predictive of later sequelae such as atrophy of the cerebellum in acute cerebellitis [9]. Finally, as previously suggested [17, 18], we hypothesized that administration of immunomodulatory therapy would be more beneficial at an earlier stage by preventing more immune‐mediated damage as opposed to letting the inflammation progress further and thereby possibly increasing the incidence of neurologic sequelae.

## 4. Conclusion

*M. pneumoniae* can cause acute postinfectious cerebellar ataxia and encephalitis/encephalopathy. Although ataxia can be self‐limited, prolonged duration of symptoms and long‐term neurological sequelae that persist for months or more are associated with both conditions. Multiple mechanisms have been suggested in the pathogenesis, but it seems that immune‐mediated damage plays a pivotal role. The children in this report improved rapidly after the administration of IVIG. The role of immunomodulatory treatment such as IVIG in decreasing the duration and severity of *M. pneumoniae* acute cerebellar ataxia and encephalitis/encephalopathy and the criteria for its administration need to be further studied.

## Author Contributions

Kathleen Ruff and Miltiadis Douvoyiannis both conceptualized and designed the study, drafted the initial manuscript, and critically reviewed and revised the manuscript.

## Funding

The authors have no funding to disclose.

## Disclosure

All authors approved the final manuscript as submitted and agree to be accountable for all aspects of the work.

## Consent

No written consent was needed from the patients as there are no patient identifiable data included in this report.

## Conflicts of Interest

The authors declare no conflicts of interest.

## Data Availability

Data sharing is not applicable to this article as no new data were created or analyzed in this study.
